# Circadian rhythms in psoriasis and the potential of chronotherapy in psoriasis management

**DOI:** 10.1111/exd.14649

**Published:** 2022-08-04

**Authors:** Andrea Luengas‐Martinez, Ralf Paus, Mudassar Iqbal, Laura Bailey, David W. Ray, Helen S. Young

**Affiliations:** ^1^ Centre for Dermatology Research and Manchester Academic Health Science Centre The University of Manchester Manchester UK; ^2^ Dr. Philip Frost Department of Dermatology and Cutaneous Surgery University of Miami Miller School of Medicine Miami Florida USA; ^3^ Monasterium Laboratory Muenster Germany; ^4^ CUTANEON Hamburg Germany; ^5^ NIHR Oxford Biomedical Research Centre John Radcliffe Hospital Oxford UK; ^6^ Oxford Centre for Diabetes, Endocrinology and Metabolism University of Oxford Oxford UK

**Keywords:** chronotherapy, circadian rhythms, psoriasis, VEGF‐A

## Abstract

The physiology and pathology of the skin are influenced by daily oscillations driven by a master clock located in the brain, and peripheral clocks in individual cells. The pathogenesis of psoriasis is circadian‐rhythmic, with flares of disease and symptoms such as itch typically being worse in the evening/night‐time. Patients with psoriasis have changes in circadian oscillations of blood pressure and heart rate, supporting wider circadian disruption. In addition, shift work, a circadian misalignment challenge, is associated with psoriasis. These features may be due to underlying circadian control of key effector elements known to be relevant in psoriasis such as cell cycle, proliferation, apoptosis and inflammation. Indeed, peripheral clock pathology may lead to hyperproliferation of keratinocytes in the basal layers, insufficient apoptosis of differentiating keratinocytes in psoriatic epidermis, dysregulation of skin‐resident and migratory immune cells and modulation of angiogenesis through circadian oscillation of vascular endothelial growth factor A (VEGF‐A) in epidermal keratinocytes. Chronotherapeutic effects of topical steroids and topical vitamin D analogues have been reported, suggesting that knowledge of circadian phase may improve the efficacy, and therapeutic index of treatments for psoriasis. In this viewpoint essay, we review the current literature on circadian disruption in psoriasis. We explore the hypothesis that psoriasis is circadian‐driven. We also suggest that investigation of the circadian components specific to psoriasis and that the in vitro investigation of circadian regulation of psoriasis will contribute to the development of a novel chronotherapeutic treatment strategy for personalised psoriasis management. We also propose that circadian oscillations of VEGF‐A offer an opportunity to enhance the efficacy and tolerability of a novel anti‐VEGF‐A therapeutic approach, through the timed delivery of anti‐VEGF‐A drugs.

## 1 INTRODUCTION

Psoriasis is a chronic, immune‐mediated skin disease that affects 1%–3% of the world population and is associated with multiple comorbidities including cardiovascular disease and metabolic syndrome.^1^, ^2^, ^3^ Psoriasis is predominantly characterised by increased keratinocyte proliferation, enhanced immune response and abnormal blood vasculature.^4^, ^5^ Biologic therapy, which targets the immune component of the disease, is one of the most effective ways to treat moderate‐to‐severe psoriasis.^6^ However, concern remains over long‐term safety, efficacy and durability of treatment response, and there is an urge to develop new treatments.^7^ The circadian system, which orchestrates all aspects of human physiology and pathology, also modulates skin functions. Research in chronobiology suggests that timing the delivery of therapy may enhance the efficacy of therapy in the management of inflammatory skin diseases such as psoriasis.^8^

**FIGURE 1:**
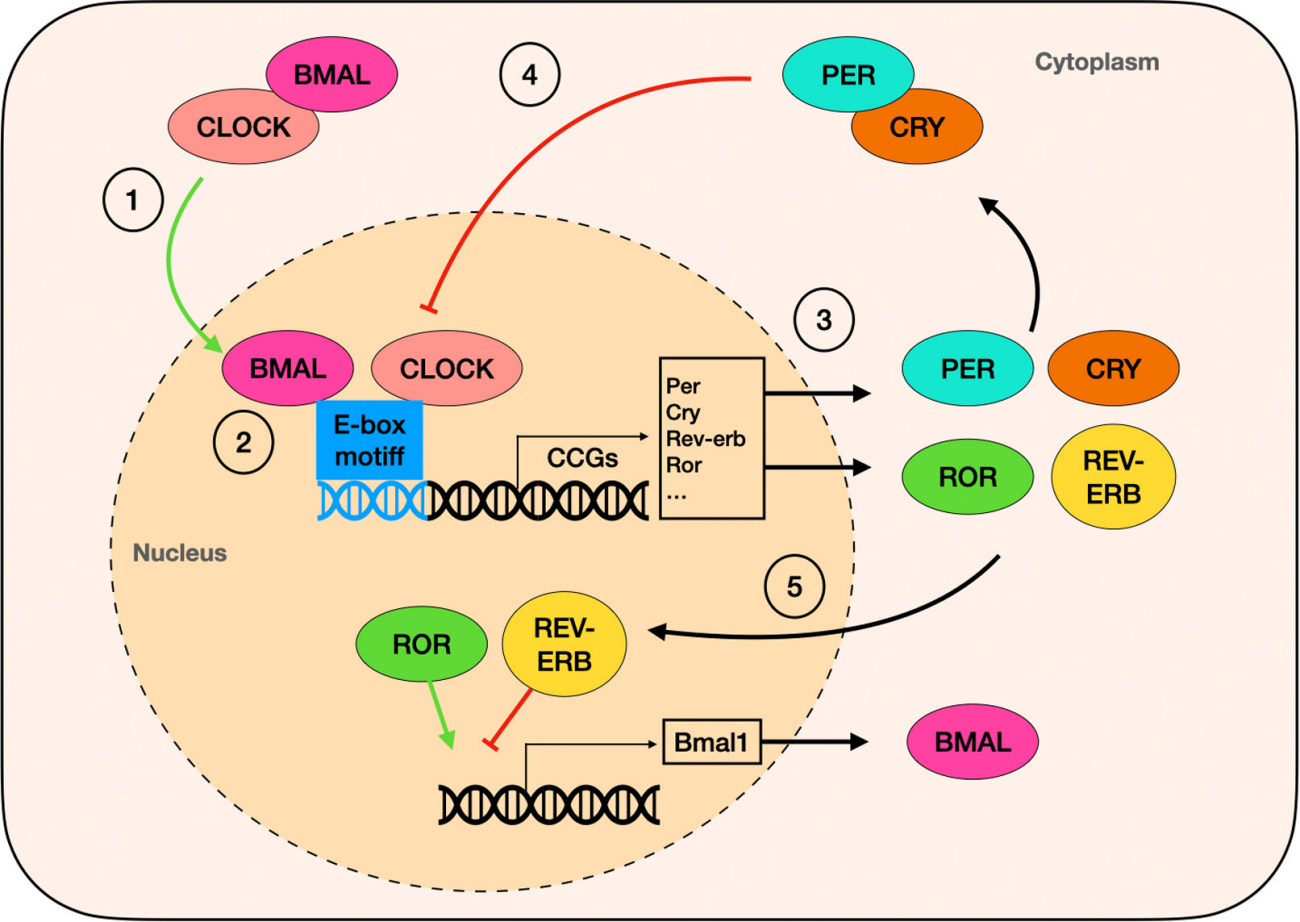
The molecular circadian system. (1) The positive arm of the feedback loop is formed by proteins of the CLOCK and BMAL families, which form a heterodimer in the cytoplasm that translocates into the nucleus. (2) where it binds to genes that contain an E‐box motif in the promoter region, also termed clock‐controlled genes (CCGs). (3) Binding of the dimer CLOCK/BMAL drives the expression of CCGs including genes of Period (PER) and Cryptochrome (CRY) families, which constitute the negative arm of the feedback response. (4) PER and CRY proteins migrate to the nucleus to block CLOCK/BMAL transcription. (5) Another auxiliary feedback loop is formed by the proteins of the REV‐ERB and ROR families. ROR and REV‐ERB proteins competitively bind to ROR elements in the nucleus, activating and repressing BMAL transcription, respectively.^8^

The circadian system consists of the master clock, which lies in the suprachiasmatic nucleus (SCN) of the brain and functions as the central pacemaker; and the peripheral clock located throughout the rest of the organs.^8^, ^9^ The master clock is entrained by light/dark cycles and is synchronised with the clocks in the different body organs and coordinated with external time cues.^10^ Individual cells within organs contain their own intrinsic transcriptional/translational loop, which constitutes autonomous body clocks that drive rhythmic tissue functions.^8^ The clock is regulated by the core clock proteins: the transcriptional activator proteins circadian locomotor output cycles protein kaput (CLOCK) and brain and muscle aryl hydrocarbon receptor nuclear translocator (ARNT)‐like protein (BMAL1); and the repressor proteins Period (PER) 1, PER2 and PER3 and cryptochrome (CRY) 1 and CRY2, which oscillate with a periodicity of approximately 24 h. In addition, there is an established transcription loop formed by two nuclear receptors, retinoic acid receptor‐related orphan receptors (ROR) and REV‐ERB, which regulate the expression of BMAL.^11^ The circadian clock core proteins modulate the expression of clock‐controlled genes, which are tissue‐ and cell‐specific (Figure 1).^12^

Genetic or environmental disruption of circadian rhythms affects various aspects of skin physiology including blood flow, transepidermal water loss, hydration and inflammation (comprehensively reviewed in Ref. [8, 12]). Circadian dysregulation is involved in various pathologies including cancer,^13^ metabolic syndrome, cardiovascular disease^14^, ^15^, ^16^ and immune‐mediated diseases such as rheumatoid arthritis^17^ and psoriasis.^18^ Angiogenesis, a key mediator of the pathogenesis of various diseases including psoriasis, is controlled by the circadian system, and maintenance of the circadian rhythms plays an important role in pathogenic angiogenesis.^19^, ^20^ Vascular endothelial growth factor A (VEGF‐A), a mediator of inflammatory angiogenesis in psoriasis, plays a major role in its pathogenesis.^21^, ^22^, ^23^ Anti‐VEGF‐A treatment strategies are not licensed for the management of psoriasis, although these are widely used in cancer and ophthalmological diseases.^24^, ^25^, ^26^ Indeed, the anti‐tumor efficacy of VEGF‐A inhibition can be enhanced by administering treatment at a time when VEGF‐A levels are at their peak. Therefore, in this viewpoint essay we will outline the role of the molecular clock in psoriasis, then focus on discussing the importance of chronotherapy in psoriasis management, before finally exploring the potential of timing the delivery of anti‐angiogenic therapy to optimise treatment response in psoriasis.

## 2 EVIDENCE OF RHYTHMICITY IN PSORIASIS INCLUDING HYPOTHESES DERIVED FROM OTHER INFLAMMATORY DISEASES

Psoriasis is classically rhythmic, and flares of disease and hallmark symptoms such as itch and pruritus manifest more severely in the evening and worsen at night.^27^, ^28^, ^29^ The circadian clock regulates many features of the immune system, and circadian disruption, whether by genetic targeting of core clock components or imposition of phase shifts in light–dark, has a significant effect on immune response.^30^ Sleep deprivation in mouse models of psoriasis led to increased levels of pro‐inflammatory cytokines, including IL‐1β, IL‐6 and IL‐12, resulting in enhanced inflammatory immune response and suggesting that circadian disruption could contribute to the pathogenesis of psoriasis.^31^ Indeed, shift work has been associated with an increased risk of developing chronic diseases such as cancer, metabolic syndrome, cardiovascular disease^32^ and psoriasis.^33^ Two studies investigating circadian fasting reported a significant reduction in Psoriasis Area and Severity Index (PASI)^34^ and significant improvement in both clinical and patient‐reported outcomes for psoriatic and inflammatory arthropathy.^35^ These findings support the idea that the peripheral and/or the central clock not only impact on the symptoms of psoriasis but may also be involved in its pathogenesis.

Studies in immune‐mediated inflammatory diseases and a small number of studies in psoriasis have determined a direct link between core components of the molecular clock and inflammatory pathways known to be relevant to psoriasis (Table 1). For instance, CRY, a transcriptional inhibitor of the TNF‐α gene, acts on a number of pro‐inflammatory target genes, regulating the expression of pro‐inflammatory cytokines, and this could link circadian disruption with the development of chronic inflammatory diseases.^36^ There are decreased expression levels of core clock genes (ARNTL, CRY2, PER1 and PER2) in lesions of psoriasis compared with those of uninvolved skin from the same patient collected at the same time, suggesting that circadian dysregulation in psoriasis plaques may contribute to the development of lesions of psoriasis.^37^

**TABLE 1:** Evidence of the link between core components of the molecular clock and inflammatory pathways in psoriasis and immune‐mediated inflammatory diseases (IMIDs)

Disease	Role of the core component of the molecular clock	Model	References
Psoriasis	CLOCK regulates psoriasis‐like skin inflammation via modulation of the expression of IL‐23 receptor in γ/δ+T cells	IMQ mouse model of psoriasis	[116]
	The relationship between the circadian clock and the skin immune response is negatively regulated by BMAL1 and can be modulated by feeding time	IMQ mouse model of psoriasis	[37]
Immune‐mediated inflammatory disease	BMAL1 regulates joint development and inflammatory arthritis in joint mesenchymal cells	Mouse model of inflammatory arthritis	[117]
	CRY regulates arthritis via pro‐inflammatory cytokine TNF‐α	Mouse model of rheumatoid arthritis	[118]
	REV‐ERBα mediates circadian regulation of innate immunity through selective regulation of the expression of pro‐inflammatory cytokine IL‐6	Mice	[119]
	REV‐ERBα controls homeostatic regulation of pulmonary inflammation via neutrophilic inflammation and increased chemokine and inflammatory cytokine responses such as CXCL5	Mouse model of pulmonary inflammation	[120]

Abbreviations: BMAL1, brain and muscle ARNT‐like 1; CLOCK, circadian locomotor output cycles kaput; CRY, cryptochrome; IL, interleukin; IMQ, imiquimod; TNF, tumor necrosis factor.

The CD4^+^ T lymphocyte, a key cellular mediator of psoriasis,^38^ possesses an intrinsic functional circadian oscillator that in response to activating stimuli such as phorbol myristate acetate (PMA)/ionomycin,^39^ or stimulation via the T‐cell receptor (TCR)^40^ drives rhythmic responses including altered cell proliferation and cytokine secretion.^17^, ^39^, ^40^ Non‐rhythmic regulatory T cells (Tregs) are driven to rhythmic activity by systemic signals such as glucocorticoid, to confer a circadian signature to chronic inflammatory arthritis.^41^ These observations suggest that rhythmic extrinsic factors regulate Treg activity within the joint. While this research was performed in a mouse model of inflammatory arthritis, it is possible that this pattern of activity is replicated in psoriasis.

Others have reported coordination of the cell cycle in epidermal stem cells by core components of the molecular clock,^42^, ^43^ suggesting that the circadian clock may contribute to keratinocyte hyperproliferation in the basal layer of psoriasis plaques. WEE1, a diurnal gene that acts to inhibit cell entry into mitosis, was down‐regulated in plaques of psoriasis, whereas other cell cycle genes such as CCNB1, UBE2C, MK167, BIRC5 and CDK1 were up‐regulated.^37^ These findings suggest that skin inflammation in psoriasis may hamper circadian control of the cell cycle in epidermal keratinocytes, which may alter keratinocyte apoptosis and keratinocyte terminal differentiation.

The peripheral clock regulates metabolism, and circadian disruption has detrimental effects on metabolic processes such as cardiovascular metabolism, lipid metabolism, and glucose metabolism.^44^ Moreover, circadian disruption is an exacerbating factor in the incidence of metabolic syndrome, which itself is associated with the risk of developing psoriasis.^17^ In addition, patients with psoriasis have aberrant circadian rhythmicity in blood pressure and heart rate, even in the absence of cardiovascular disease per se.^45^, ^46^ Hormones such as melatonin and glucocorticoids, which are key controllers of the circadian rhythms, may also be involved in the pathogenesis of psoriasis.^47^ For instance, glucocorticoids, which may be used to treat inflammatory diseases such as psoriasis, exert pleiotropic effects on the immune system.^48^ While endogenous glucocorticoids drive diurnal oscillations in T‐cell function under physiological conditions,^49^ exogenous glucocorticoids and stress‐induced glucocorticoids have anti‐inflammatory and immunosuppressive effects. Indeed, they suppress pro‐inflammatory cytokine expression such as IFN‐γ production by T helper (T_H_) 1 cells and cell‐mediated immunity such as exhaustion of CD8^+^ T cells.^50^, ^51^ Patients with psoriasis have lost the nocturnal peak of melatonin and the usual circadian rhythm of melatonin secretion.^47^ Low melatonin levels in psoriasis were associated with pruritic episodes and elevated TNF‐α, IL‐6 and IL‐8.^52^ The immunomodulatory properties of melatonin are well known,^53^ and modulation of melatonin levels could be used to regulate the release of pro‐inflammatory cytokines in psoriasis.

Taken together, these findings support the concept that circadian disruption is involved in the pathogenesis of psoriasis. Moreover, circadian dysregulation in patients with psoriasis may influence key symptoms of the disease. Despite the evidence supporting a role for the circadian system in psoriasis, the dominant cell type driving circadian disease expression in psoriasis remains undetermined. Understanding circadian modulation of key regulatory pathways involved in skin inflammation is essential for a full understanding of the pathogenesis of psoriasis. In turn, this may lead to the identification of key circadian targets for therapeutic intervention and enable targeted and personalised timing of psoriasis therapy to maximise treatment efficacy.

## 3 THE AUTONOMIC NERVOUS SYSTEM, INFLAMMATION AND THE CLOCK

Inflammation in diseases such as psoriasis is under multiple levels of circadian control including the intrinsic timing system of the immune cells and regulation via the autonomic nervous system. The autonomic nervous system, composed of sympathetic (SNS) and parasympathetic (PNS) branches, has extensive crosstalk with the immune system. In response to stimuli, sympathetic neurons release catecholamines (predominantly noradrenaline), which activate adrenergic receptors in the target tissue. For instance, lymphoid organs show extensive innervation by the SNS, allowing the SNS to directly influence immune cells via adrenergic receptor activation, from which an immune cell may be activated to a pro‐inflammatory or anti‐inflammatory state dependent on the environmental cues.^54^

Autonomic nervous outputs are under circadian control, following the entrainment of the central circadian clock (SCN) by the light/dark cycle. For example, the SCN sets the rhythmic synthesis and secretion of catecholamine neurotransmitters within the SNS, both in peripheral tissues at nerve terminals and in the bloodstream released by the adrenal gland.^55^ Abnormalities in the cutaneous cholinergic and adrenergic system, which exhibit circadian rhythmicity through the circadian fluctuation of acetylcholine and catecholamine release, have been reported in psoriasis and may influence its pathogenesis.^56^, ^57^, ^58^

## 4 CHRONOTHERAPY

The increasing understanding of circadian rhythms has led to its translation into clinical practice and the development of the field of chronotherapy, which aims to improve the tolerability and efficacy of treatments. Chronotherapy can minimise the toxicity and side effects through timing drug delivery with the oscillatory behaviour of drug and treatment targets in disease tissues, including rhythmic changes in drug absorption, distribution, metabolism and excretion.^59^ Surgical procedures can also be chronomodulated. For instance, afternoon surgery in patients undergoing aortic valve replacement leads to better outcomes compared with morning surgery, due to the circadian variations that underlie perioperative myocardial injury.^59^, ^60^

Studies using a murine model of psoriasis‐like inflammation demonstrated that the expression of the vitamin D receptor (VDR) exhibits diurnal variation with VDR levels peaking in the middle of the active period (night‐time for nocturnal animals such as mice).^61^ Using this model, the authors demonstrated that the efficacy of topical vitamin D analogues, one of the first‐line treatments for psoriasis, was enhanced when administered during early to middle active period compared with dosing during early to middle inactive period.^61^ A recent study demonstrated greater efficacy of topical corticosteroids when applied to plaques of psoriasis in the evening.^62^ Similar observations come from several small clinical studies, in which administration of corticosteroids for the treatment of asthma^63^ and rheumatoid arthritis^64^ in the afternoon or evening is more efficacious than in the morning. For instance, the administration of low doses of glucocorticoids with a relative short biological half‐life before the circadian flare, defined as the increase in IL‐6 synthesis, improves acute rheumatoid arthritis symptoms such as the duration of morning stiffness and joint pain, among others.^64^ One study reported that circadian rhythms modulate the skin's ability to cope with ultraviolet (UV) B damage in mice and suggested that UV radiation exposure should be restricted to the morning hours in humans.^65^ Studies investigating the influence of the circadian rhythms on UV therapy are missing, and therefore, chronotherapy in the field of UV therapy remains speculative although worthy of future investigation. Clinical trials for cancer treatment demonstrate the beneficial effects of chronotherapy.^66^, ^67^, ^68^, ^69^, ^70^, ^71^ Chronotherapy remains underexplored in psoriasis, and most of the research has been performed in other diseases. Evidence of successful application of chronotherapy in other diseases such as inflammatory‐mediated diseases and cancer suggests that chronotherapy is also possible for psoriasis.

Identification of psoriatic‐specific changes in gene expression rhythmicity could contribute to the development of chronotherapeutic strategies for psoriasis. Understanding the circadian fluctuations in keratinocyte proliferation or inflammation would also be informative. Linking these changes to candidate drugs, with short half‐lives, and optimising drug delivery timing to coincide with the time when their target gene is most active could lead to improved treatment efficacy. Candidate drugs with short half‐lives are most appropriate for chronotherapy as timing of administration has a greater impact on their action. For instance, the optimal administration time for short half‐live statins is before bedtime, as cholesterol synthesis peaks during the night.^10^ Low‐dose methotrexate (<30 mg/m^2^), one of the traditional systemic agents used for psoriasis, has a short half‐life ranging from 3 to 10 h^72^ and is potentially an ideal candidate for chronotherapy. Methotrexate chronotherapy has enhanced therapeutic effectiveness for rheumatoid arthritis compared with conventional treatment scheduling.^73^

Other key cytokines that could be appropriate targets for chronotherapy include TNF‐α, IL‐23 and IL‐17A.^10^ For instance, TNF‐α expression presents circadian fluctuation in patients with rheumatoid arthritis^74^ and it has been shown that timing the administration of methotrexate based on circadian cycling of TNF‐α levels determines treatment efficacy in mouse models of arthritis.^75^ Chronotherapy based on the rhythmic oscillation of TNF‐α, IL‐23 and IL‐17A expression deserves further exploration.

## 5 EXPLOITING THE CIRCADIAN CLOCK TO PERSONALISED ANTI‐ANGIOGENIC THERAPY IN PSORIASIS

The peripheral clock regulates the human vasculature and angiogenesis.^76^, ^77^ Circadian fluctuation of VEGF‐A production and VEGF‐A mRNA expression has been demonstrated in zebrafish embryos^20^ and in tumor cells.^78^ Moreover, VEGF‐A daily oscillations are coordinated with BMAL1 expression levels, which is directly involved in circadian clock‐regulated angiogenesis.^20^ Disruption of the clock by genetic manipulation of core clock genes or constant exposure to light led to dysregulated angiogenesis in zebrafish embryo,^20^, ^79^ suggesting that circadian disruption leads to vascular pathology.

Other authors reported that the levels of VEGF‐A mRNA in murine tumor cells increase in response to hypoxia and they fluctuate in a circadian pattern, with VEGF‐A levels peaking during the light phase and decreasing during the dark phase. While BMAL1 positively regulated VEGF‐A production under hypoxic conditions in mice, PER2, CRY1^78^ and DEC2 negatively regulated its production.^80^ Moreover, the anti‐tumor efficacy of the anti‐angiogenic agent SU1498 (a VEGFR‐2 tyrosine kinase inhibitor) was enhanced when it was administered during the early light phase rather than during the early dark phase in mice.^78^ These findings corroborate other studies where decreased efficacy and increased side effects are reported when anti‐angiogenic therapy is delivered at night.^81^ The increased sensitivity to anti‐angiogenic therapy during the light phase may be at least partly attributed to increased VEGF‐A levels during the light phase, suggesting that circadian variations in VEGF‐A production affect the efficacy of anti‐angiogenic therapy.^78^ Since mice are nocturnal animals, VEGF‐A levels peaking during the light phase correspond to the increase in VEGF‐A levels in humans or zebrafish, which are diurnal, during the night.

Mice deficient in ROR‐α, a key regulator of BMAL1,^82^ exhibit increased induction of angiogenesis following tissue ischaemia. While it has not been demonstrated that the disruption in BMAL1 signalling is the cause of ischaemia‐induced angiogenesis, these findings suggest that ROR‐α may be a negative regulator of ischaemia‐induced angiogenesis.^83^ The regulation of VEGF‐A mRNA and VEGF‐A protein expression levels by the circadian fluctuations of HIF‐α has been confirmed in a human colon cancer cell line following serum shock.^84^ Indeed, studies using human cell lines identified BMAL2 and CLOCK as key regulators of VEGF‐A, with levels of VEGF‐A peaking during the night.^85^, ^86^ It has also been reported that the level of VEGF‐A in the plasma of patients with POEMS (polyneuropathy, organomegaly, endocrinopathy, monoclonal protein and skin changes) increases at night and decreases during the day.^87^ This circadian pattern of expression of VEGF‐A protein is mirrored by variation in response to several inhibitors of angiogenesis according to the time of their administration.^88^ Preclinical evidence demonstrates that the antitumor efficacy of anti‐angiogenic agents can be enhanced by administering treatment at a time when VEGF‐A levels are peaking.^78^ These investigations on circadian regulation of VEGF‐A and angiogenesis, which have been mostly performed in the field of cancer, suggest that VEGF‐A may be an ideal candidate for chrono‐targeted therapy.

Angiogenesis plays a central role in psoriasis,^89^ largely mediated by VEGF‐A,^90^ which is overexpressed in the skin and plasma of patients with psoriasis.^91^, ^92^, ^93^ The VEGF‐A/VEGF receptor pathway constitutes a potential therapeutic target for psoriasis management.^21^ VEGF‐A overexpression in mice led to the development of skin lesions that share many psoriasis features,^94^, ^95^, ^96^ and targeting VEGF‐A in mouse models of psoriasis resulted in psoriasis clearance.^94^, ^95^, ^97^, ^98^, ^99^, ^100^, ^101^ Indeed, conventional treatments for psoriasis down‐regulate angiogenesis and reduce VEGF‐A levels as part of their therapeutic effect.^102^, ^103^, ^104^ This work is further discussed and can be accessed in Ref. [21, 105].

Anti‐VEGF‐A therapy has not been licensed for psoriasis, but there are seven case reports of patients who have experienced psoriasis improvement while receiving anti‐VEGF‐A therapy for the treatment of cancer.^106^, ^107^, ^108^, ^109^, ^110^, ^111^, ^112^, ^113^ For instance, two patients experienced psoriasis improvement after bevacizumab (Avastin®, a monoclonal antibody to VEGF‐A) treatment (administered intravenously).^106^, ^107^ Two cases of patients who experienced significant psoriasis improvement after treatment with sunitinib (Sutent®, a VEGF‐A receptor tyrosine kinase inhibitor) have been reported (administered orally).^108^, ^109^ There are three cases of patients who experienced significant psoriasis improvement after sorafenib (Nexavar®, a VEGF‐A receptor tyrosine kinase inhibitor) treatment (administered orally).^110^, ^111^, ^112^

**FIGURE 2:**
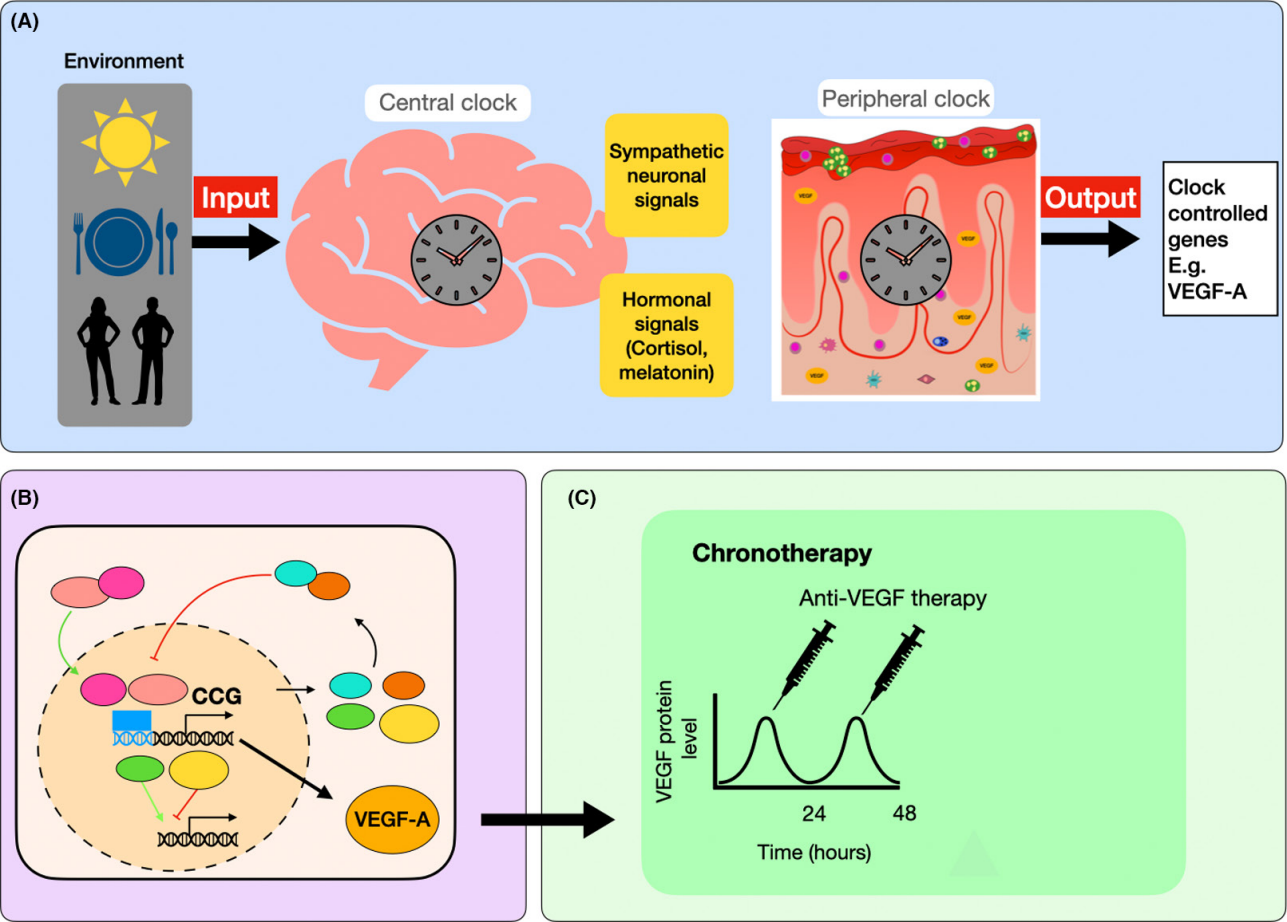
Model of anti‐angiogenic therapy with a chronotherapy approach for psoriasis management. (A) The light input received at the suprachiasmatic nucleus along with other external stimuli such as food intake and social interactions is sent to the peripheral clocks via endocrine and neural signals to synchronise the external stimuli with the biological rhythms throughout the body.^11^ In the skin, there is an intrinsic circadian clock that regulates skin functions in the different skin compartments including the vasculature.^12^ (B) At the molecular level, the core clock proteins control the transcription of several genes termed clock‐controlled genes, and induce their rhythmic transcription. Evidence showed that VEGF‐A expression follows an oscillatory rhythm controlled by the circadian system.^78^ (C) Anti‐VEGF‐A therapy could be enhanced by administering the drug at a time when VEGF‐A protein levels are peaking (CCGs, clock‐controlled genes).

These investigations led to investigating the utility of VEGF‐A inhibitors in human skin. We have shown in preliminary studies that VEGF‐A blockade reduces blood vessel density in psoriasis skin ex vivo (unpublished data). Moreover, in the previous work we identified a group of patients with psoriasis genetically predisposed to produce high levels of VEGF‐A, who are associated with early‐onset psoriasis and a severe disease phenotype.^114^, ^115^ This subgroup of patients may be more likely to benefit from an anti‐angiogenic therapy.^114^, ^115^ Therefore, our investigations are focused on VEGF‐A as a key chronotherapeutic target. We hypothesise that rhythmic oscillations in the expression of VEGF‐A could be used to enhance the efficacy of anti‐angiogenic therapy in psoriasis (Figure 2). Delivering anti‐VEGF‐A treatment at a time when VEGF‐A levels are peaking in the plasma and skin of patients with psoriasis may enhance the efficacy of anti‐VEGF‐A therapy in psoriasis and could help minimise toxic effects. More studies are required to elucidate the role of VEGF‐A rhythmicity in psoriasis and to determine the chronotherapeutic potential of anti‐angiogenic therapy.

## 6 TESTING THE HYPOTHESIS

In order to investigate the potential of chronotherapy in psoriasis, identification of the circadian factors specific to immune cell populations such as T cells and non‐immune cell populations such as keratinocytes and endothelial cells in psoriasis is required. The psoriasis‐specific changes in gene expression rhythmicity and their link to candidate drugs need to be defined. In vitro models or ex vivo human skin models could be used to investigate circadian regulation in psoriasis and test the efficacy of circadian therapies.

## 7 WHY IT MATTERS

Psoriasis represents a model of chronic inflammation with visual and accessible pathology, which is amenable to serial sampling and investigation. Determining how the clock couples to inflammatory angiogenesis in the skin of patients with psoriasis, as well as the involvement of the clock outputs in the pathogenesis of psoriasis, is likely to produce novel therapeutic strategies for the management of psoriasis and other inflammatory diseases.

## 8 CONCLUSIONS

We postulate psoriasis pathogenesis is driven by the circadian clock and that timing the delivery of current treatments, and novel therapies such as anti‐VEGF‐A agents, could enhance their effectiveness in patients with psoriasis.

The cutaneous microvasculature, which is circadian‐rhythmic, plays a key role in the pathogenesis of psoriasis. VEGF‐A mediates blood vessel abnormalities in psoriasis, and there is evidence that the expression of VEGF‐A fluctuates in a circadian pattern. Thus, the effectiveness of VEGF‐A blockade may be influenced by the circadian clock, with a window of maximal and minimal responsiveness to VEGF‐A blockade. Understanding how the circadian clock regulates VEGF‐A oscillations in the skin of patients with psoriasis has the potential to inform chronotherapeutic approaches. As the skin vasculature underlies prominent chronobiological controls, adequate timing of the delivery of anti‐angiogenic therapy should optimise the treatment response.

## AUTHOR CONTRIBUTIONS

HSY designed the study. ALM, RP, MI, LB, DWR and HSY wrote the manuscript. All authors contributed to the writing/editing of the manuscript and have read and approved the final version of the manuscript.

## CONFLICT OF INTEREST

The authors have no conflicts of interest to declare.

## Data Availability

Data sharing not applicable to this article as no datasets were generated or analysed during the current study.
